# Quality microbiological diagnostics and antimicrobial susceptibility testing, an essential component of antimicrobial resistance surveillance and control efforts in Pacific island nations

**DOI:** 10.5365/wpsar.2018.9.3.004

**Published:** 2020-02-20

**Authors:** John Kenneth Ferguson, Jacklyn Joseph, Samson Kangapu, Hilda Zoleveke, Nicola Townell, Trevor Duke, Laurens Manning, Evelyn Lavu

**Affiliations:** a School of Biomedical Sciences and Pharmacy, University of Newcastle, Callaghan, Australia.; b Port Moresby General Hospital, Port Moresby, Papua New Guinea.; c Infectious Disease Department, Sunshine Coast University Hospital, Queensland, Australia.; d National Referral Hospital, Honiara, Solomon Islands.; e Centre for International Child Health, Department of Paediatrics, University of Melbourne, Australia.; f School of Medicine, University of Papua New Guinea, Port Moresby, Papua New Guinea.; g School of Medicine, University of Western Australia, Harry Perkins Research Institute, Fiona Stanley Hospital, Western Australia, Australia.; h Central Public Health Laboratory and University of Papua New Guinea, Port Moresby, Papua New Guinea.

## Abstract

**Problem:**

Emerging bacterial antimicrobial (antibiotic) resistance (AMR) is a global threat to human health. However, most lower income countries do not have microbiological diagnostic testing for prompt, reliable confirmation of bloodstream infection and identification of AMR.

**Context:**

Clinicians in Pacific island nations are increasingly challenged by patients who have infection due to antimicrobial-resistant bacteria. Treatment of infection remains empirical because of a lack of diagnostic testing capacity and may follow guidelines that were formulated without reference to local measures of AMR prevalence. There is limited understanding among clinicians of microbiology testing and test interpretation.

**Action:**

Examine the lessons learnt from pilot laboratory development programmes in two Pacific island nations that focused on establishing standard procedures for micrological diagnostics and antimicrobial susceptibility testing (AST) and on improving the training of clinicians to increase their use of laboratory services.

**Outcome:**

The pilot programmes addressed a range of logistical difficulties and evaluated two blood culture systems. They also examined and improved internal QC implementation and evaluated the prevalence of AMR.

**Discussion:**

Continued development of microbiological diagnostic capability in the Pacific region is paramount. Pacific Island nations need to develop the capability of at least one central laboratory to culture AMR pathogens and subject them to quality-controlled AST or arrange for suitable referral to a nearby country.

**Discussion:**

This study demonstrated a persistently high prevalence of three major bacterial STIs across four countries in WHO’s Western Pacific Region during nearly two decades. Further strengthening of strategies to control and prevent STIs is warranted.

## PROBLEM

Antimicrobial resistance (AMR) is a major threat to human health. (*1*, *2*) Patients with sepsis who are treated with an antimicrobial for which the causative pathogen is non-susceptible have an increased risk of mortality. (*2*) The World Health Organization (WHO) Global Antimicrobial Surveillance System prioritizes the following bacterial pathogens, commonly associated with resistance, for surveillance: *Mycobacterium tuberculosis, Escherichia coli, Klebsiella pneumoniae, Shigella, Salmonella, Neisseria gonorrhoeae, Acinetobacter baumannii, Pseudomonas aeruginosa, Staphylococcus aureus* and *Streptococcus pneumoniae*. (*3*) Resistance to these pathogens is associated with poor response to treatment, prolonged hospitalization and excess mortality. (*4*)

Knowledge of bacterial antimicrobial susceptibility testing (AST) patterns is the cornerstone of an effective clinical and public health response to AMR. Reliable AST results are decision-making support tools that enable clinicians to prescribe appropriate antibiotics for patients. As a public health tool, AST data that describe the prevalence, geographic distribution and temporal trends of resistant pathogens should inform standard treatment guidelines that are developed in the Pacific region.

A lack of laboratory infrastructure and microbiological expertise in many Pacific island nations has made AMR surveillance unreliable. Most published data are based on studies from major hospitals without details of quality control (QC), (*5*, *6*) or the testing standard used. (*7*) In addition, external to the laboratory, pre-analytical factors such as proper specimen selection and collection are often deficient. Reliably sensitive blood culture systems are generally unavailable, preventing effective diagnosis of severe bacterial infection and greatly impeding AMR surveillance. (*8*)

This manuscript aims to examine the importance of quality bacterial culture and antimicrobial susceptibility for clinician guidance and for effective AMR surveillance. It highlights important AST concepts that are perhaps poorly understood by laboratory and clinical staff in many Pacific island nations. It examines lessons learnt from pilot laboratory development programmes based in Solomon Islands and Papua New Guinea (PNG) and discusses recent AMR surveillance data from Port Moresby General Hospital in PNG.

## CONTEXT

Citizens of Pacific island nations are particularly vulnerable to the consequences of AMR. The burden of infection, in both the community and the hospital, is high, and appropriate treatment of antimicrobial-resistant infections often require prolonged treatment with expensive antibiotics that may not be available. Infection with resistant organisms results in longer hospital stays that increase the risk of further complications such as nosocomial infection (e.g. health care–associated pneumonia). Many hospitals in this region have limited infection prevention and control systems, and multiresistant organisms are frequently spread by health-care staff with unclean hands and fomites (e.g. contaminated, reused equipment, stethoscopes, clothing) and through contact with contaminated environments.

In many Pacific island nations, antibiotic use during health-care attendances is high, and common antibiotics may be purchased without prescription at markets. Unregulated antibiotic use in the agricultural sector occurs. Contamination of stream and tank water with enteric bacteria, including *Salmonella* Typhi, is documented within the region. (*9*) Increasing international travel provides another risk factor for AMR acquisition and spread. Taken together, these factors promote the emergence, acquisition and transmission of antimicrobial-resistant pathogens.

WHO has facilitated the development of national AMR plans that emphasize the importance of regional laboratories (serving human and animal health sectors) that are able to reliably perform bacterial culture and AST supported by an AMR reference laboratory that orchestrates QC measures and operates an external quality assurance (EQA) programme. (*3*)

### How does quality microbiological culture and AST enable better patient care?

Where health literacy is low and infection rates are high, patients often present with severe infection due to delayed presentation and neglected co-morbidities. Effective diagnostic testing is an essential addition to the clinical assessment. Culture and AST of the infecting pathogen allow the clinician to modify treatment to adequately target a pathogen and ensure effective, definitive antimicrobial therapy. This improves patient outcomes and reduces mortality. (*10*) Therapeutic changes may involve moving to a narrow spectrum antimicrobial (e.g. benzylpenicillin or flucloxacillin) for susceptible isolates or using a broader spectrum agent when culture and AST demonstrate a resistant organism (e.g. extended-spectrum β-lactamase [ESBL; ceftriaxone resistant] producing *Klebsiella* or related species or methicillin [flucloxacillin-resistant *Staphylococcus aureus* [MRSA]).

### What are the meanings of minimum inhibitory concentration and clinical breakpoint?

Due to the lack of medical microbiologists and limited scientific awareness of microbiology in most Pacific island nations, there is value in explaining these concepts. The minimum inhibitory concentration (MIC) indicates the particular antimicrobial concentration (mg/L) that is required to inhibit growth of the infecting organism in the laboratory. The MIC test is performed by exposing a bacterial isolate inoculated into broth to decreasing dilutions of the antimicrobial. After incubation, the broth well with the lowest antimicrobial concentration without bacterial growth (i.e. clear) becomes the MIC value. There are two standards organizations, the Clinical and Laboratory Standards Institute (CLSI) (*11*) and the European Committee of Antimicrobial Susceptibility Testing (EUCAST), (*12*) that specify use of the same international MIC testing reference standard (International Standards Organization 20776–1). (*13*) By considering a wide range of laboratory and clinical evidence for each bacterial species and antibiotic, each organization defines an MIC value that represents the clinical breakpoint that divides isolates into susceptible and non-susceptible (resistant) categories. If an isolate tests as non-susceptible to an antimicrobial, then treatment of a clinical infection with that drug is likely to fail. The clinical breakpoints determined by the two organizations may differ for the same antibiotic and organism combination because of differing processes of deliberation. It is important to specify the standard in use and reference the updates to clinical breakpoint tables that are published by EUCAST and CLSI every January. Clinical breakpoints are also set for other AST methods such as disc susceptibility testing derived by comparison with the ISO standard MIC test.

### How is AST usually performed?

**Figure 1:**
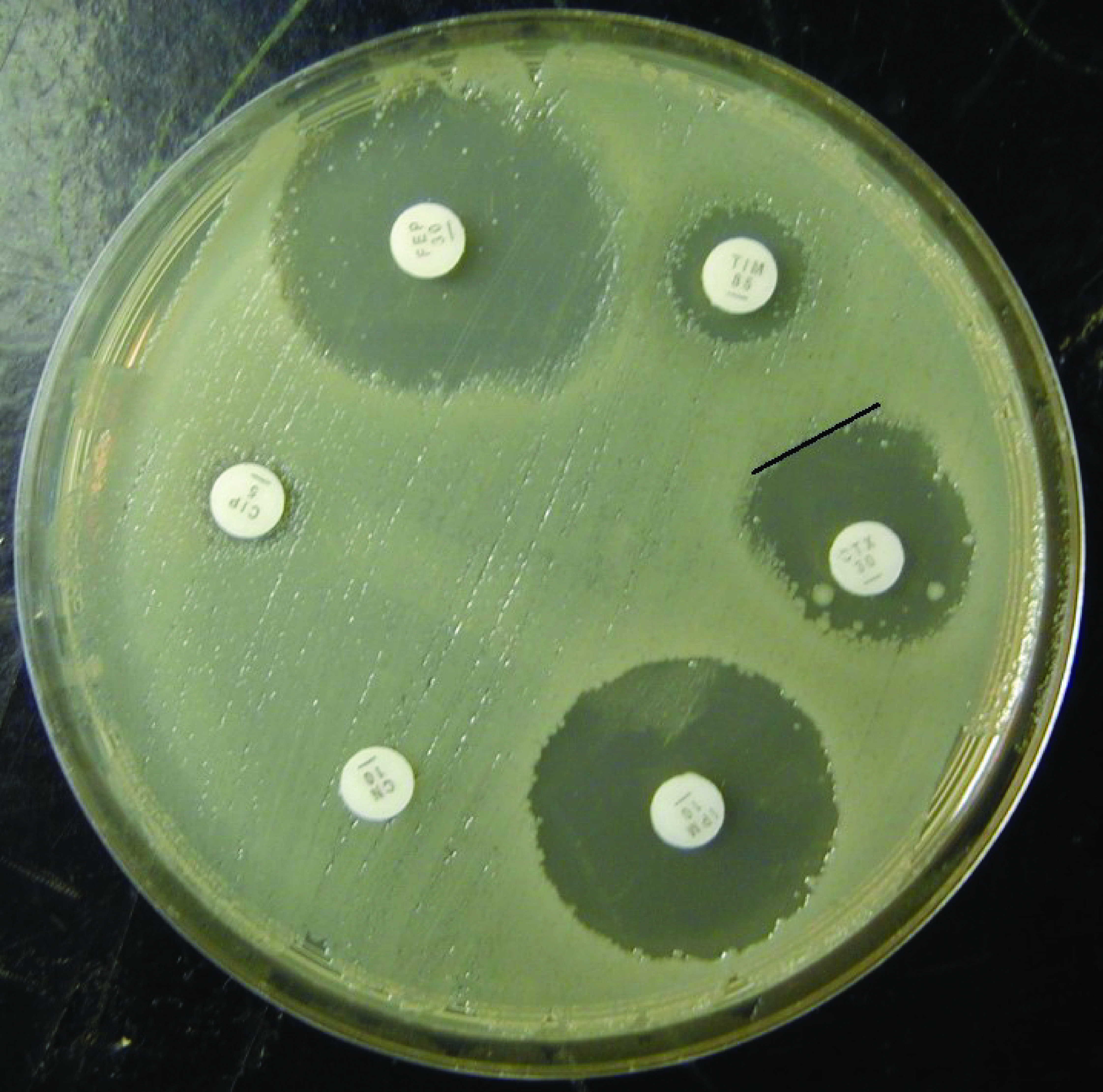
**Antimicrobial susceptibility test using the disc method**

Disc susceptibility is the most commonly used method in the Pacific region (**Fig. 1**). A disc containing a quantity of antimicrobial is placed onto an agar plate that has been seeded with the organism in question. After 18 hours of incubation, the antibiotic will inhibit growth of the organism resulting in a zone of absent growth. If the measured zone of inhibition is larger than the prespecified clinical breakpoint zone size, this indicates that the organism can be reported as susceptible to that drug.

[**insert** Figure 1]

### What challenges occur with AST?

There are many variables that affect the AST result, potentially causing error (e.g. a resistant isolate being reported as susceptible). The agar growth medium requires preparation with consistent quality of ingredients and thickness, and each batch requires QC for sterility and its ability to support growth of control organisms. Blood-containing media are required for some species (e.g. *Streptococcus pneumoniae* and *Haemophilus influenzae*), which remains problematic where required sheep or horse blood is unavailable. Expired human blood is therefore used widely in the Pacific region; however, this is not an appropriate substitute. The incubation temperature and duration must be correct. Antimicrobial discs must be in-date, of the right strength and stored correctly to avoid degradation. Discs from certain manufacturers have variable quality, highlighting the need to obtain them from reliable sources. (*14*) The technician reading the AST must ensure that the disc inhibition zone is measured and interpreted correctly.

## ACTION

Laboratory capacity development in PNG (at Port Moresby General Hospital [PMGH]) and in Honiara, Solomon Islands by the Pacific Region Infectious Diseases Association has focused on improving the training of scientists and establishing standard procedures for the microbiology service. Commercial blood culture systems have been introduced to increase positive culture detection and reduce the turnaround time (reduced from 5 days to 2 days). Clinician education concerning the effective use of blood cultures, AMR and its detection has been conducted as a prelude to the introduction of hospital antimicrobial stewardship.

Internal QC systems have been introduced to provide assurance that AST results are correct. All antimicrobial discs are tested against QC bacterial strains weekly. If the tested zone size is within a specified range, then the process is controlled and the laboratory can issue a valid result. Both laboratories participate in EQA to regularly test unknown isolates from a reference organization.

## OUTCOME

### Logistics

A range of issues have had to be addressed, including ordering and supply of consumables and availability of serviced incubators and fridges. The electricity supply has not been a limiting factor in these sites to date.
Regular visits and teleconference support to train and mentor technical and scientific staff and assist with supervision by the in-charge pathologist have been useful.
Development of standard operating procedures, in collaboration with scientific staff, and more general quality management systems have also been important. The WHO approach to stepwise implementation of laboratory quality management systems has been useful.
Sheep or horse blood for production of blood-containing agar media is generally unavailable. Most laboratories are substituting expired, donated human blood. However, such media usually fail to grow important organisms such as *Streptococcus pneumoniae* and *Neisseria gonorrhoeae*.

### Commercial blood culture systems

Evaluation of the automated blood culture system BACTEC (Becton Dickinson Co., Franklin Lakes, NJ, USA) at PMGH from 2016 to 2017 revealed blood culture detection rates for significant pathogens of 8.4% in adults and 6.0% in children. These rates were significantly higher than those obtained with in-house media and pathogens were isolated more rapidly. The 2018 data from Solomon Islands using the BacT/ALERT® (bioMerieux Co., Norwest NSW, Australia) system found a detection rate of 5.8%.
Contamination rates were too high (adults, 7%; children, 15%) at PMGH and 8.8% in Honiara, Solomon Islands, highlighting the need for further effort and training to improve specimen collection practices.

### Internal QC implementation

Challenges included identification and maintenance of a −80 °C freezer for QC bacterial strain storage and implementation of correct handling procedures for master and working cultures of QC strains.
Maintaining scientific supervision of QC testing to ensure it is properly performed regularly and that results are formally reviewed and acted on.
QC troubleshooting: An important example was that of gentamicin susceptibility at PMGH, where the zone size was persistently below the expected range. A new batch of gentamicin discs showed a correct zone size, indicating that the existing discs, although in-date, had lost potency and needed replacement.

### Prevalence of AMR

The 2018 PMGH cumulative antibiogram showed rates of methicillin-resistant *Staphylococcus aureus* (MRSA) of 39% to 60% across all groups (adult and paediatric, community and hospital locations). MRSA susceptibility for doxycycline and co-trimoxazole remained high. Samoa and East Timor have also recorded high rates of MRSA. (*7*, *15*) The 2017 data from Honiara, Solomon Islands indicated low rates of MRSA (2% of 53 isolates).

At PMGH, 63% and 25% of Gram-negative (*E. coli, Klebsiella* and related species) isolates showed non-susceptibility to ceftriaxone in inpatients and outpatients, respectively, indicating high levels of ESBL. Ceftriaxone non-susceptible isolates were usually also non-susceptible to ciprofloxacin and gentamicin, and a minority were susceptible to chloramphenicol. ESBL isolates are also prevalent in New Caledonia, Fiji and East Timor. (*7*, *15*, *16*) The 2017 data from Honiara found non-susceptibility to ceftriaxone to be 15% (200 tested isolates).

## Discussion

Continued development of microbiological diagnostic capability and reliability is paramount for clinicians and AMR surveillance. Owing to the unavailability of appropriate media, culture and AST of *N. gonorrhoeae* and *S. pneumoniae* are rarely performed. Each Pacific island nation needs to develop the capability for at least one central laboratory to culture these pathogens and subject them to quality-controlled AST or arrange for suitable referral to a nearby country.

MRSA is common in Pacific island nations, resulting in bone, joint, lung or blood-stream infections. (*6*, *15*) While inclusion of vancomycin on essential medication lists has cost and logistical implications, it needs to be considered for management of proven MRSA bacteraemia and empirical management of severe sepsis. Standard treatment guidelines will require revision, and clinicians will require education concerning the use and monitoring of vancomycin.

Rates of ESBL across South-East Asia and the Pacific region nations have been high for more than 10 years and have been associated with sustained hospital, neonatal and intensive care outbreaks with high mortality. (*4*) Community carriage is common and is exacerbated by overuse of antibiotics and a lack of infection control resulting in hospital patient-to-patient transmission and subsequent nosocomial infection. Where local data indicate high rates of ceftriaxone resistance, this agent cannot be relied upon for empirical use in situations where Gram-negative pathogens predominate (e.g. urinary, intra-abdominal, biliary sepsis, nosocomial infection). Meropenem, a carbapenem class of β-lactam antibiotic, that is not widely available in Pacific island nations, is the recommended first-line option for treatment of ESBL bloodstream infections. (*17*) In line with recent WHO recommendations, meropenem should be incorporated into national essential drug lists. When meropenem becomes available, clinician education and restrictive measures will be required to ensure appropriate prescribing. Overuse of meropenem leads to the emergence of meropenem-resistant, Gram-negative species (carbapenemase-producing *Enterobacterales* [CPE]). CPEs have very limited treatment options due to multiresistance and are now prevalent in many world locations, especially in Asia. Pacific island nations will need to prepare for CPE emergence, including the ability to reliably detect CPE in laboratories.
